# Microfluidic System for Observation of Bacterial Culture and Effects on Biofilm Formation at Microscale

**DOI:** 10.3390/mi10090606

**Published:** 2019-09-12

**Authors:** Xiao-Yan Zhang, Kai Sun, Aliya Abulimiti, Pian-Pian Xu, Zhe-Yu Li

**Affiliations:** 1 School of Environment, Harbin Institute of Technology, Harbin 150090, China; 2 State Key Laboratory of Urban Water Resource and Environment, Harbin Institute of Technology, Harbin 150090, China

**Keywords:** microfluidics, biofilm, continuous flow, antibiotic, microscale, *E. coli*

## Abstract

Biofilms exist in the natural world and applied to many industries. However, due to the variety of characteristics caused by their complex components, biofilms can also lead to membrane fouling and recurrent infections which pose threats to human health. So, to make the best use of their advantages and avoid their disadvantages, knowing the best time and methods for improving or preventing biofilm formation is important. In situ observation without fluorescence labeling in microscale and according to a time scale is useful to research biofilm and confine its formation. In this study, we developed a microfluidic system for real-time observation of bacteria culture and biofilms development at microscale. We cultured *E. coli* ATCC 25922 on a chip at continuous flow of the velocity, which could promote bacterial formation. Biofilms formation under the condition of adding amoxicillin at different times is also discussed. In addition, the mixed strains from sludge were also cultured on chip, and possible factors in biofilm formation are discussed. Our results show that a microfluidic device could culture microorganisms in continuous flow and accelerate them to adhere to the surface, thereby promoting biofilm formation. Overall, this platform is a useful tool in research on initial biofilm formation, which can contribute to preventing biofouling and infections.

## 1. Introduction

Biofilm is secreted by bacteria and widely exists in nature. It aggregates free-floating microorganisms to form a sessile community, which enables bacteria to inhabit the surface. The biofilm is one of the most important factors leading to membrane fouling in membrane bioreactors (MBRs) in water treatment industry [1]. However, on the other hand, biofilms comprising microorganisms grown on carriers can remove some micropollutants and biological phosphate [2,3]. In order to optimize the positive effects and minimize the negative effects, actions should be taken at initial stage to promote or inhibit biofilm formation. Therefore, it is essential to study biofilm formation at time and spatial scales.

In the process of biofilm formation, changes occur in genotype and phenotype in spatial scales. At nanoscale, planktonic bacteria communicate and coordinate with each other by enzymes and molecular signals. At the microscale, bacteria adhere to the solid surface and aggregate to form microcolonies, which secrete extracellular polymeric substances (EPS) for building biofilms structures. At the mesoscale, mature biofilms can be observed and used in practical application at the macroscale [4]. Biofilms at mesoscale and macroscale are strongly associated with practical applications, so most studies focus on the factors of biofilm development at these scales. However, intensive studies of effects on the initial formation process at microscale are also important, which are the best stage to facilitate or inhibit the biofilm formation. In a traditional shake flask, most bacteria are in the planktonic state, so the biofilms are hard to culture [5]. Culturing biofilms by shake flask is not only labor-intensive, but also the characteristics of biofilms formed cannot remain the same. The reason is that the bacterial heterogeneity of spatial distribution [6] leads biofilms to vary widely in physiological conditions [7] and cellular physiologies [8]. Moreover, because biofilms are settled in different regions, mass transfer passing through the biofilm cannot be controlled. All the above factors result in different biology characteristics.

Microfluidic technology can bear the organisms to culture at microscale space, and the cultural conditions can be precisely controlled. For example, Hogan et al. prevented *S. aureus* biofilm formation on a plastic biochip which was modified by polyurethane [9]. With the remarkable advantage of rapid analysis [10], low cost [11], low sample consumption [12], high throughput [13], controllable conditions [14], microfluidics has been a promising method for bioanalysis. In the forming process of biofilms, real-time observation is crucial to obtain the information of morphology at microscale. Undoubtedly, the microfluidic system based on a miniaturized chip is the best method to study the initial biofilm formation and the effects on the biofilms imposed by environmental conditions, including nutrient concentration [15], surface properties [16], shear stress [17], quorum sensing [18]. In the meanwhile, microfluidic device is able to confine the bacterial growth in micrometers and to mimic the practical environmental conditions to probe the complex interplay at different time scales [19,20]. Recently, researchers focus microfluidic system coupled with different detection modes to study biofilms. Funari et al. monitored microbial biofilm formation by nanoplasmonics in situ [21]. Li et al. studied biofilm accumulation and adhesive strength in microfluidics coupled with a microscope [22].

In this work, we designed and fabricated microfluidic chips for bacteria culture at continuous flow and real-time investigation on the initial formation of biofilm at microscale. To determine the hydrodynamic factor of biofilm formation, *E. coli* ATCC 25922 were cultured on the chip as model bacteria at different velocities. We also studied the influence of adding amoxicillin at different stages on initial biofilm formation with the microfluidic device. In addition, we analyzed the biofilm formation of mixed strains, which were cultured from the sludge of secondary sedimentation tank. The influence of amoxicillin on mixed microorganisms with biofilms was also discussed.

## 2. Materials and Methods

### 2.1. Materials and Reagents

Luria Bertani (LB) broth are composed of 0.5% yeast extract, 1% peptone, 1% NaCl and deionized water, all of which were purchased from Beijing Ao Xing Bio-tech (Beijing, China). Ethanol and amoxicillin were obtained from Sigma-Aldrich (Shanghai, China). SU-8 2050 negative photoresist was purchased from Micro Chem Corp. (Newton, MA, USA). Polydimethylsiloxane (PDMS) Sylgard 184 was obtained from Dow Corning (Midland, MI, USA). Borosilicate glass was achieved from Matsunami (Kyoto, Japan). FilmTracer TM SYPRO^®^ Ruby Biofilm Matrix Stain was purchased from Invitrogen (Waltham, MA, USA).

### 2.2. Sample Preparation

*E. coli* ATCC 25922 with flagella was used as a model strain in this work. *E. coli* BL 21 as control was purchased from the China Center of Industrial Culture Collection (CICC). Prior to culture on the chip, bacteria from a LB agar Petri dish were transferred to LB medium and incubated overnight. *E. coli* was diluted in LB medium to obtain OD_600_ in the range of 0.112–0.115, then injected into chip to culture. The mixed strains were obtained from sludge of a secondary sedimentation tank; 1 mL suspended sludge were incubated in pretreated piggery wastewater for 24 h. Then the mixed strains were diluted in pretreated piggery wastewater to obtain OD_600_ in the range of 0.112–0.115 and used to culture on chip.

### 2.3. Bacteria Culture and Biofilm Formation in Microfluidic Device

The microfluidic device was firstly sterilized by flushing with 75% alcohol overnight and washed by deionized water in sequence. After ultraviolet (UV) sterilization for 30 min, diluted *E. coli* in LB medium was injected into the culture chamber at the constant flow rate of 5 μL/min by syringe pumps. The fresh medium was continuously injected into the chamber after balancing for 2 min, so the free flow bacteria was washed away. Fewer bacteria were able to adhere to the glass, then they grow under the continuous flow at the rate of 0.5 μL/min and form the biofilm. Bacteria were cultured on the chip at room temperature of 25 ℃, which was controlled by constant temperature and humidity air conditioner. The temperature nearby microfluidic system was monitored by the electronic thermometer in the whole experiment, and the temperature difference was ±1 °C. The antibiotics were added in different periods of bacterial growth. For bacteria from sludge on chip culture, the medium was pretreated piggery wastewater by high-temperature sterilization.

### 2.4. The Process of Biofilm Stain on Chip

Bacteria were cultured on chip under the continuous flow at the rate of 0.5 μL/min. After 11 h, water was injected into the culture chamber and LB medium was flowed away. On account of LB medium interfering with fluorescent staining, we injected water. Then SYPRO Ruby was injected into the culture chamber at the rate of 0.5 μL/min. At the continuous flow, the samples were incubated for up 1 h, protected from light. In the end, the samples were observed by an inversion microscope.

### 2.5. Microfluidic Device Fabrication

The standard soft lithographic process was used to fabricate this chip. The depth of channel and chamber was 50 μm. PDMS prepolymer and curing agent by the ratio of 10:1 were mixed and degassed, then poured onto a SU-8 mold. After baking at 70 °C in the oven for 2 h, the PDMS mold was peeled off and bonded with glass. In order to avoid the organic and metallic contamination, the borosilicate glass was sequentially cleaned in following solutions, H_2_SO_4_:H_2_O_2_ (4:1), H_2_O:H_2_O_2_:NH_4_OH (50:10:1), H_2_O:H_2_O_2_:HCl (5:1:1). After washing, the glass plate was immersed in H_2_O/H_2_O_2_/NH_4_OH solution (7:2:1) to increase the hydrophily. The PDMS layer and the glass layer were bounded by using UV O-zone treatment (Model 42-UVO-Cleaner, Jelight, Irvine, CA, USA) for 5 min and baking for 70 °C for 15 min.

### 2.6. Microfluidic System and Optical Image Analysis

**Figure A1:**
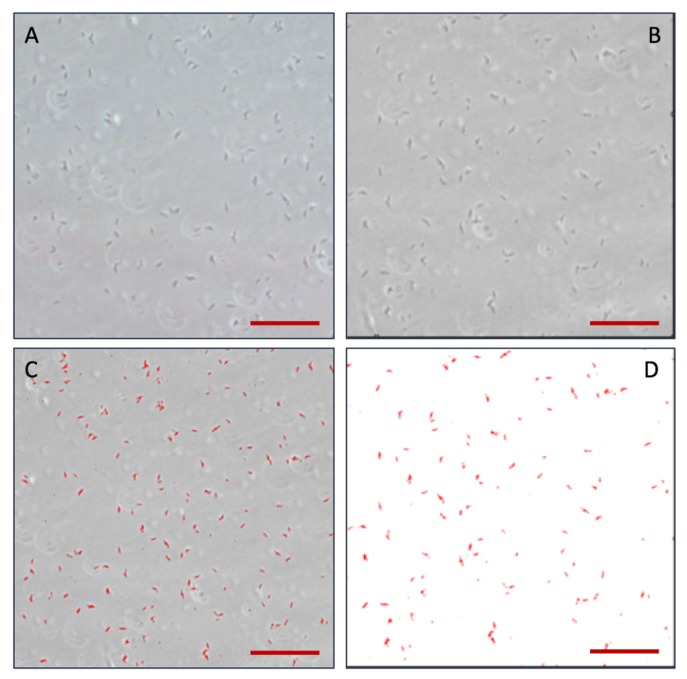
Image processing: (**A**) original image; (**B**) the RGB image was transformed into grey format image; (**C**) image after subtracting background; (**D**) binary image. The scale bar is 40 μm.

This system consisted of one chip to culture bacteria; two pumps (AL-1000, World Precision Instruments, Sarasota, FL, USA), two syringes (Gastight 1001, Hamilton, Reno, NV, USA) and one three-way valve (MV201, LabSmith, Livermore, CA, USA) to control the fresh medium and antibiotic. The optical images were acquired by an inversion microscope (Olympus, IX 71, Tokyo, Japan) with a charge coupled device (CCD) camera (Olympus, DP80, Tokyo, Japan). The images of bacteria magnified 400 times were taken every hour, and were analyzed by Image J software. The steps of image processing and the number of cells were stated in Figure A1.

In suspension culture, the growth rate is determined by optical density (OD_600_). For the bacterial culture on a chip, bacteria are able to adhere to the glass and grow in a monolayer. Hence, bacterial mass is proportional to superficial area and increment ratio R = S_i_/S_0_ [23]. In this study, we use the relative growth rate ln R to quantify the speed of bacterial growth.

## 3. Results and Discussion

### 3.1. Chip Design and Operation

**Figure 1:**
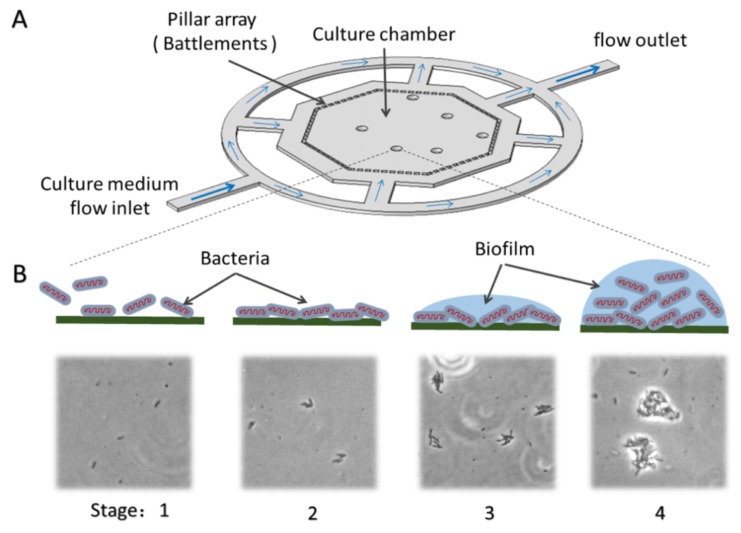
Schematic illustrations of chip structures for bacteria culture and biofilm formation. (**A**) The diameter of inscribed circle of regular octagon area is 2 mm. The size of a single micropillar is 54 μm ×40 μm (top view) and the gap between pillars is 30 μm. The depth of both the channel and the culture chamber is 50 µm. (**B**) Schematic illustrations and optical images of stages of biofilm development: (1) Loose adhesion to the surface. (2) Irreversible attachment. (3) Bacteria secrete the extracellular polymeric substances (EPS) to form the biofilm structure, and the biofilm matures. (4) Biofilms are completely mature.

We developed a PDMS chip to culture bacteria and form biofilm under continuous flow. The process of bacterial culture formation was observed by an inversion microscope. The detailed design of the chip structure is shown in Figure 1A, and the schematic diagram of biofilm formation is shown in Figure 1B. This design was aimed to culture bacteria and form biofilms by reducing fluidic impact and supporting steady culture conditions. The flow from the inlet was separated by annular channels, which was used to keep mean velocity inside of the octagon chamber. The inner octagon area is designed as the culture chamber, and surrounded by the battlements arrays of micro pillars, the top view of each pillar is 54 µm × 40 µm, and the gap between the two neighboring pillars is 30 µm. Moreover, the pillars could also prevent the gas bubble from entering into the culture chamber. The width of all the channel is 200 µm, the depth of both the channels and the chamber is 50 µm.

The sample of diluted bacteria should be loaded into the chip through the outlet of the culture chamber. After balancing for 2 min, the medium was injected continuously from the inlet of the culture chamber. Due to the asymmetric structure, this injection mode enables bacteria to distribute randomly in the culture chamber. In order to flow the planktonic bacteria in chip, the medium was injected at the 10 μL/min for 5 min, then the velocity was reduced to 0.5 μL/min.

### 3.2. Bacterial Adhesion and Biofilm Formation on the Chip

In this study, we challenged to culture *E. coli* as model microorganism to observe biofilm formation on the chip. Bacteria biofilms are difficult to culture and obtain in traditional shake flasks, although nearly all kind of bacteria secrete viscous substrates to form biofilms. At present, few studies have investigated the microbe except for *Pseudomonas Aeruginosa* (*P. Aeruginosa*), which is a model microorganism. Because the pili of *P. Aeruginosa* contribute greatly to its ability to adhere to the surface and form biofilm easily. Due to this characteristic, minimal biofilm eradication concentration (MBEC) of *P. Aeruginosa* could be measured in most studies [24]. However, it is hard to obtain the biofilms of *E. coli* in laboratory by traditional methods. Taking advantage of the large special surface area of chip, bacteria are able to adhere to the chip wall and grow to form the biofilm easily.

*E. coli* is with peritrichous flagella, which propel bacteria forward by rotation motion under the control of the flagella bundle [25]. Under the action of the thrust force generated by flagella, *E. coli* is difficult to be confined on chip in liquid. For on chip culture, the medium was injected after bacteria loading, so free-floating bacteria can be washed away and the bacteria with the slime-encased substances can adhere to the glass wall.

**Figure 2:**
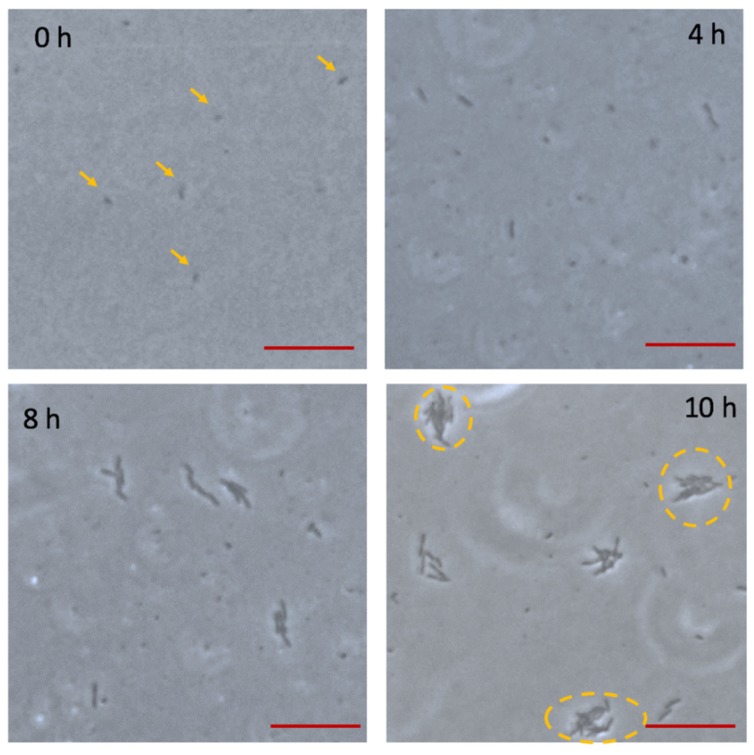
The photomicrograph of bacteria culture on chip in continuous flow at 0 h, 4 h, 8 h, 10 h. At 0 h, bacteria distribute in monolayer. At 4 h, bacteria adhere to the surface and grow. At 8 h, semitransparent substances surrounding the bacteria begin to form. At 10 h, bacteria grow into multilayers, and biofilms can be observed obviously. The scale bar is 5 μm.

**Figure 3:**
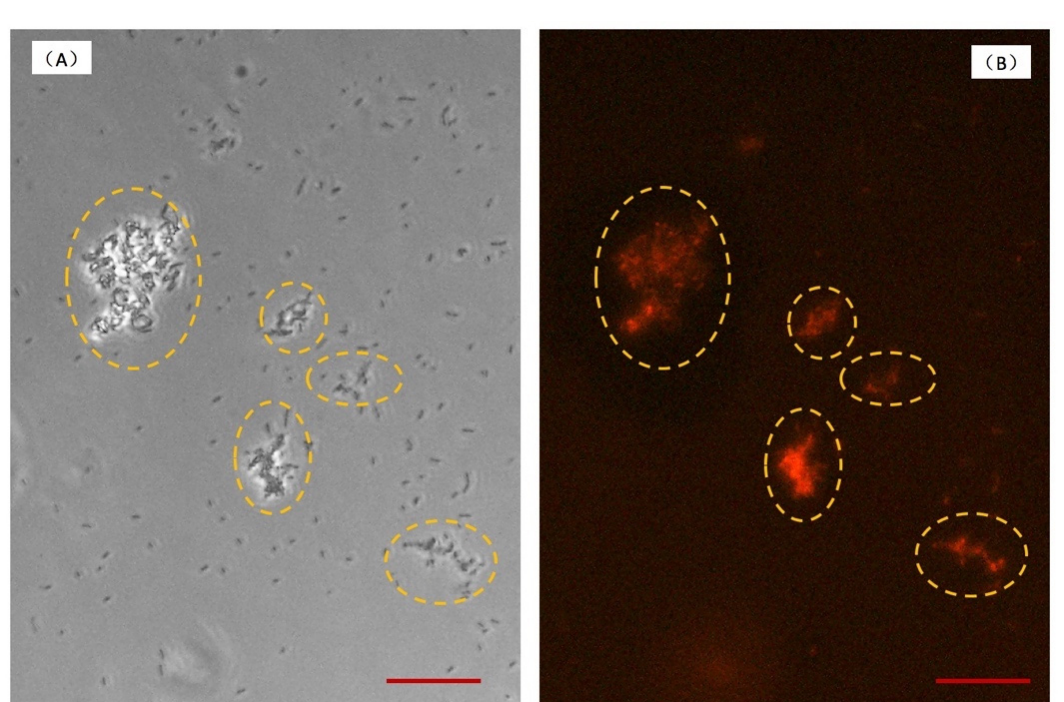
The image of biofilms in bright field (**A**) and in fluorescence (**B**). After 11 h culture, biofilms were stained by SYPRO Ruby in culture chamber. The scale bar is 20 μm.

As shown in Figure 2, only a few microorganisms were observed adhering to the glass wall and were confirmed to distribute in a monolayer at 0 h. With the continuous flow passing through the adherent bacteria, sufficient nutrient can be supplied, and at the same time metabolites were taken away. In addition, bacteria of loose adhesion can be flowed away by the continuous flow. After 4 h incubation, bacteria irreversibly adhered to the surface, which is presented in Figure 2. For 8 h culture, bacteria colonies were formed and kept in a monolayer. During this period, semitransparent substances began to occur in periphery of colonies. At 10 h, with bacterial growth, a single colony became larger, and bacteria grew into multilayers. In the meantime, the semitransparent substances enclosing the colonies could be observed obviously. According to the micrographs, the process of bacteria growth and biofilm formation is consistent with the distinct stages of biofilm life cycle [26]. Firstly, planktonic bacteria loose adhere to the surface, and this process is reversible. Secondly, bacteria begin to undergo division, and adhesion is irreversible. Thirdly, bacteria secrete the EPS, which is a significant component of biofilm. Fourthly, the biofilms approach maturely. After 11 h culture, the crystal materials with three-dimensional structure obviously appeared, which is shown in Figure 3A. According to bright filed observation, the bacteria colonies were wrapped by crystal materials. In order to verify the crystal materials are biofilms, we stained the biofilms and observed in fluorescence. SYPRO Ruby stain labels most classes of proteins, which are the component of biofilms. The fluorescence image is in Figure 3B, which shows the location of crystal materials forming in a bright field (Figure 3A) are consistent with fluorescence image. However, for long term biofilm formation, fluorescence observation couldn’t be persistent. We studied biofilms in the bright field instead of fluorescence, which contributes to end-point observation.

### 3.3. The Effect of Velocity on Bacteria Growth and Biofilms Formation

High shear rates or an extended period of time exposed to strong shear force, which was generated by velocity, may lead to bacterial death [27]. However, under no shear force, bacteria will grow in a planktonic condition instead of sessile communities. Therefore, it is necessary for the on-chip culture to choose an appropriate velocity of constant flow, which can promote bacteria to form biofilms and avoid bacteria death. The shear rate of bacteria in the microfluidic chip was calculated by

$$\gamma =\frac{Q}{wh^{2}}$$

where *γ* is shear rate, *Q* is flow rate, *w* is the width of channel, *h* is the depth of channel [28].

**Figure 4:**
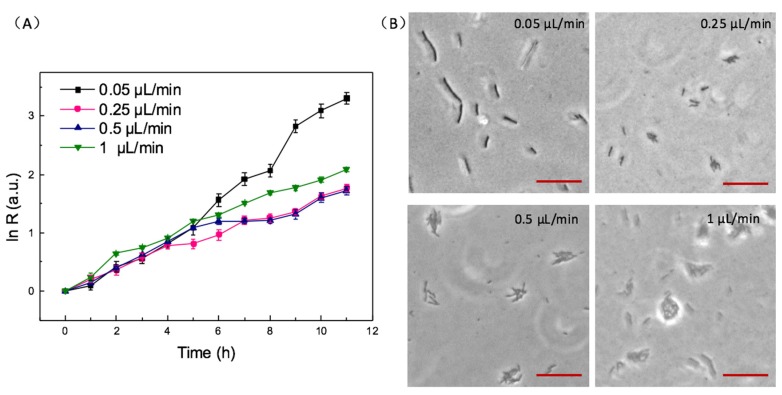
Growing situations at continuous flow of different velocities. (**A**) bacterial growth rate during incubation at velocity of 0.05 μL/min, 0.25 μL/min, 0.5 μL/min, 1 μL/min. (**B**) The photomicrographs of bacteria culture on chip for 10 h at velocity of 0.05 μL/min, 0.25 μL/min, 0.5 μL/min, 1 μL/min. The scale bar is 5 μm. Error bars are standard deviations.

Bacteria were cultured at different velocities of 0.05, 0.25, 0.5, 1.0 μL/min, and the results are shown in Figure 4A. The shear rate is 1.67 s^−1^, 8.33 s^−1^, 83.3 s^−1^, 166.6 s^−1^, respectively. For 7 h culture, the bacterial growth rate at 0.05 μL/min is much larger than other higher velocities. It illustrates that the velocity of 0.05 μL/min cannot flow away free-floating bacteria and the bacteria of loose adhesion to the surface, so the bacteria are cultured at the condition of almost no shear force. At the velocity of 0.05 μL/min, bacteria grow in the planktonic state instead of in biofilms. It is proved in Figure 4B that after 10 h incubation, bacteria do not form communities at 0.05 μL/min. At 0.25 and 0.5 μL/min, the bacterial growth rates are similar, and lower than the velocity of 1 μL/min. Because shear stress can promote bacteria adhering to and dispersing on the interface at the initial stage of forming biofilms [29]. With larger velocity of continuous flow, bacteria suffered higher shear stress. It leads to faster biofilm formation and higher bacteria growth rate. As shown in Figure 4B, bacteria could form communities at 0.25, 0.5, 1 μL/min. However, it is obvious that bacteria have grown into multilayers at 1 μL/min at 10 h, which implies biofilms are more mature than other cases. The multilayer bacteria were caused by high velocity, which leads to the failure of counting. Therefore, the velocity of 1 μL/min is not suitable for the investigation of the influence factors on biofilm formation. Finally, we selected 0.5 μL/min for the following experiments.

### 3.4. The Effects of Antibiotic on Biofilm Formation during the On-Chip Growth

Based on mass transport, the process of biofilms formation occurs in three characteristic time scales: (1) hydrodynamic process. During this period, momentum balance is achieved in seconds by momentum transfer including viscous convection and dissipation. (2) Mass transport solute process. During this period, the substrate mass balance is achieved in minutes by substrate diffusion. (3) Growth process. During this period, the biomass balance is achieved in hours or days in biomass growth scale [30]. In this report, on the basis of different characteristics in the process of biofilms formation, we studied the effects of adding amoxicillin at different stages of the bacteria growth.

**Figure 5:**
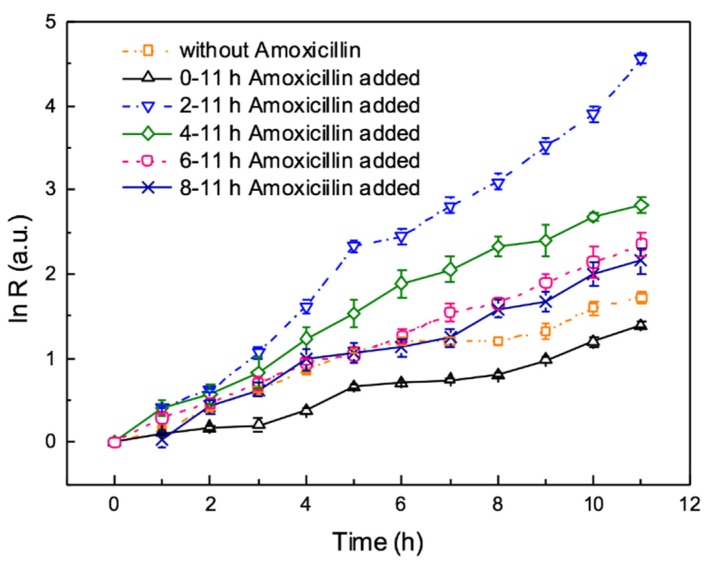
The effects of antibiotic on biofilm formation during the on-chip culture. 4 mg/L amoxicillin were added after 0, 1, 3, 5, 7 h incubation with continuous flow at the velocity of 0.5 μL/min. Error bars are standard deviations.

**Figure A2:**
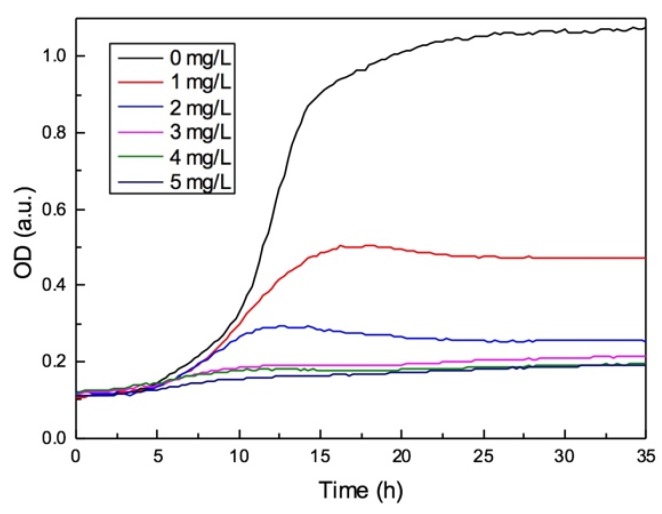
The growth curve of bacteria in 96 well-plate. *E. coli* were inhibited by 1, 2, 3, 4, 5 mg/L amoxicillin.

Bacteria were injected into the culture chamber and cultured for 0, 2, 4, 6, 8 h respectively, then 4 mg/L amoxicillin was started to be added continuously. According to the bacterial growth curves, the minimal inhibition concentration (MIC) of *E. coli* ATCC 25922 to amoxicillin is 4 mg/L, which is shown in Figure A2. Therefore, we studied the effects of 4 mg/L amoxicillin on the process of biofilm formation. Amoxicillin is a kind of beta-lactam antibiotic, preventing cell wall from synthesis to inhibit bacteria division, leading bacteria to barely grow. The curves of the bacteria growth rate are shown in Figure 5. Adding the antibiotic at the beginning (black solid line), bacteria growth rate is the lowest. Compared to the bacterial growth curve without amoxicillin, it is obvious that bacterial growth is inhibited by amoxicillin, which was added at the beginning. However, the inhibition did not last during the whole process. The bacterial growth curve shows the growth rate approaches to zero in the first 3 h. After 3 h, bacteria began to grow slowly. This is mainly because bacteria loosely adhere to the glass. Though a small amount of bacteria can adhere the glass, viscous substrates surrounding them are rare, and the antibiotic is easily permeated to affect bacteria. With the viscous substrates increasing and biofilm forming, antibiotic is absorbed to the biofilms so that bacteria in them can grow. After 4 h, under the protection of biofilms, bacteria begin to grow.

It is very interesting that the result of adding amoxicillin after 1 h incubation is completely different from the above description, the bacteria growth rate is larger than all the other cases including with adding amoxicillin and without adding amoxicillin. It illustrates that biofilms are formed in the initial stage. After 1 h culture, amoxicillin was added, at this time the biofilms outside of the bacteria are able to resistant to it. According to the previous report, antibiotics can stimulate the bacteria to release EPS, which belong to the major construction material of biofilms, leading to membrane fouling of bioreactors [31,32]. EPS consists of polysaccharides, proteins and other macromolecules, such as DNA, lipids, which is like a biological glue immobilizing bacteria to attach to and aggregate to the surfaces. Relying on EPS, biofilms possess strong viscoelasticity and adhesive strength [33]. Compared to 4, 6, 8 h amoxicillin added, bacterial growth rate increases rapidly after adding antibiotics. This is principally because EPS protect bacteria from flowing away. From the growth curves of amoxicillin adding at different periods, it illustrates that the bacterial growth rate increases rapidly with adding amoxicillin earlier. Therefore, adding antibiotics earlier will shorten the time of loose adhesion.

### 3.5. Effect of Antibiotic on Mixed Microorganism by On-Chip Culture

In aforementioned experiments, single strain bacteria have been cultured on chip and the effects of biofilm formation studied. However, biofilms existing in natural environment are formed by sessile communities of microorganisms. Therefore, we use bacteria from sludge of secondary sedimentation tank for on-chip culture and biofilm observation.

**Figure 6:**
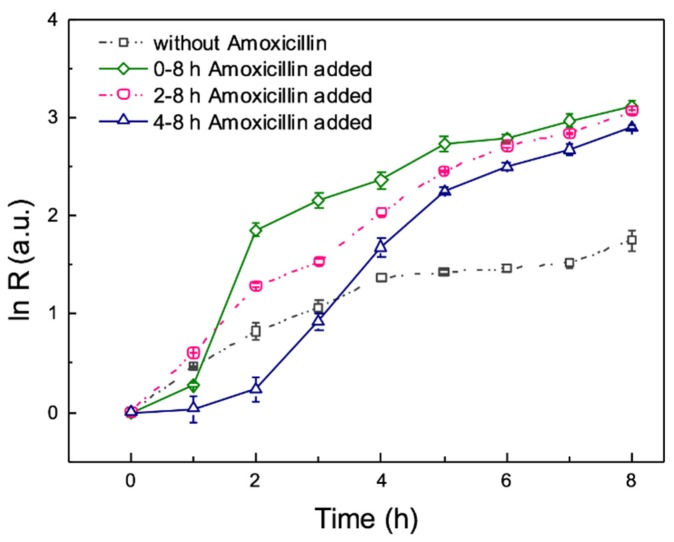
Effect of antibiotic on mixed microorganism for on-chip culture. Mixed strains microorganism from the sludge of secondary sedimentation tank were cultured in pretreated piggery water, and 4 mg/L amoxicillin were added after 0, 1, 3 h incubation. Error bars are standard deviations.

The medium for mixed microorganism culture was pretreated piggery wastewater, which was sterilized before loading into the chip. The results of 8 h on-chip culture are shown in Figure 6. By comparing curves between pure bacteria (orange line in Figure 5) and mixed microorganism (black line in Figure 6), it is obvious that the growth rate of mixed strains increases larger than single strain. For on-chip culture under continuous flow, growth relies on biofilms, which can immobilize and protect the microorganism. Mixed microorganism are more likely to form biofilms, leading to microorganism attach to the surface rapidly. The main reason is that some strains of the microorganism in the sludge is regulated by genes to form biofilms [34]. In addition, cell-cell communication can promote bacteria to form biofilms [35]. The cell-to-cell communication mechanism is known as quorum sensing (QS), which allows bacteria to communicate by direct cell-to-cell contact or extracellular messenger molecules [18]. For example, quorum sensing in *P. aeruginosa* can upregulate the secretion of EPS. Hence, the growth rate of mixed strains increases more rapidly than a single strain under the conditions of gene expression and quorum sensing.

Similar to the above experiment, mixed microorganisms were loaded, then culture medium with 4 mg/L amoxicillin was added into the culture chamber at 0, 2, 4 h. The total culture time was 8 h, because biofilms have formed multilayers. As we mentioned, counting bacteria becomes difficult in multilayers. In contrast to single strain of bacteria, it is obvious that mixed microorganisms are not influenced by the antibiotic (black line in Figure 6). This illustrates that some strains in mixed microorganisms are resistant to amoxicillin. At the same time, the growth rate of mixed microorganisms on a chip increases rapidly under the influence of the antibiotic. As shown in Figure 6, the growth curves are close after amoxicillin added at 0, 2, 4 h. This is because the colonies aggregate and multilayers bacteria leads to large errors in counting. Therefore, biofilms of mixed microorganisms approach maturely earlier than the single stain.

In a natural environment, microorganisms are existed in community instead of single strain. Consequently, the biofilms formation are influenced by various factors, such as QS and antibiotics. Especially in membrane bioreactors, there are complex reasons for membrane fouling caused by EPS has and this takes a few days or even longer to study the influence factors. This platform provides a rapid method to observe the biofilms formation influenced by single or multiple factors.

## 4. Conclusions

We have developed a novel microfluidic system to observe biofilm formation under continuous flow. The platform provided a simple and fast way at microscale to study the effects on the bacterial biofilm formation, including hydrodynamic factor, antibiotic and quorum sensing. The chip is very useful for bacterial culture, and enables bacteria to adhere to the surface and form biofilm. In the continuous flow, free-floating bacteria were washed away, and weak shear force could promote biofilm formation. We also found that amoxicillin adding into the culture chamber at different periods had different effects on the *E. coli* adhesion and biofilm formation. Different phenomena were observed when using mixed microorganisms from the sludge of the secondary sedimentation tank. Complicated factors including quorum sensing and antibiotics have to be considered in this case. All the above experiments indicated that the microfluidic platform is a prospect tool to study the biofilms influenced by single or multiple factors for rapid observation at microscale. The future potential application of this system can be valuable for controlling biofilm, such as in clinics or the water treatment industry.
